# Initial Clinical Experience With Robotic Liver Resection Using the Saroa Surgical System: Practical Feasibility and Technical Challenges

**DOI:** 10.1111/ases.70337

**Published:** 2026-06-23

**Authors:** Nobuhisa Tanioka, Satoru Seo, Kento Shinno, Kazune Fujisawa, Masaya Munekage, Hiromichi Maeda, Hiroyuki Kitagawa

**Affiliations:** ^1^ Department of Surgery, Kochi Medical School Kochi University Nankoku Japan

**Keywords:** hepatectomy, laparoscopic surgery, minimally invasive surgery

## Abstract

**Introduction:**

The Saroa Surgical System is a newly developed robotic platform with force‐feedback capability and a compact, relatively low‐cost design. This study aimed to report our initial clinical experience with robotic liver resection using the Saroa Surgical System, with particular emphasis on its feasibility, safety, practical advantages, and the technical challenges encountered during the early implementation phase.

**Methods:**

This retrospective case series included the first eight consecutive patients who underwent Saroa‐assisted robotic liver resection at our institution between November 2024 and August 2025. The selected patients had superficial liver lesions that did not require anatomical resection. Clinical characteristics and short‐term perioperative outcomes were descriptively analyzed.

**Results:**

Eight patients underwent partial hepatectomy: five had hepatocellular carcinoma, two had metastatic liver tumors, and one had intrahepatic cholangiocarcinoma. The median operative time, console time, hepatic inflow occlusion time, and blood loss were 267 min, 136 min, 41 min, and 17.5 mL, respectively. Three patients required unplanned conversion to laparoscopy, which was performed smoothly using a port configuration compatible with laparoscopic surgery. One patient who underwent a third hepatectomy developed postoperative bile leakage (Clavien–Dindo grade IIIa). The median length of postoperative hospital stay was 10 days.

**Conclusion:**

Robotic liver resection using the Saroa Surgical System was feasible in selected patients during the introductory phase. Its compatibility with the laparoscopic workflow, compact design, and force‐feedback capability may offer practical advantages. However, the restricted working range, limited instrument availability, and relatively low grasping force remain as limitations.

## 1 Introduction

Laparoscopic liver resection for liver malignancies offers several advantages over open liver resection, including reduced postoperative complications and shorter hospital stay [1, 2]. However, owing to its reliance on straight instruments, laparoscopic liver resection remains technically demanding, particularly for lesions located in the posterosuperior segments [3, 4], and requires a substantial learning curve [5]. Robotic liver resection, represented by the da Vinci system, is being increasingly adopted because it provides three‐dimensional visualization, tremor filtration, articulated instrument movement, and improved precision, thereby expanding the role of minimally invasive liver surgery [6].

Despite these advantages, conventional robotic surgery lacks force feedback, and excessive force transmission to tissues may result in unintended collateral injury [7]. In addition, the high installation and operating costs of robotic systems remain major barriers for small‐ to medium‐sized hospitals, raising important concerns regarding healthcare economics [8]. The Saroa Surgical System (Riverfield Inc., Tokyo, Japan) is a newly developed surgical robotic platform designed to address these issues. Unlike previous systems, it incorporates real‐time force‐feedback capability through a pneumatically actuated mechanism and has been developed as a compact, lightweight, and relatively low‐cost platform. The system received manufacturing and marketing approval in Japan in May 2023.

This study aimed to describe our initial clinical experience with robotic liver resection using the Saroa Surgical System, with particular emphasis on its feasibility, safety, practical advantages, and the technical challenges encountered during the early implementation phase.

## 2 Methods

### 2.1 Study Design and Patient Selection

This retrospective observational case series evaluated the safety and feasibility of a liver resection program using the Saroa Surgical System. This study included the first eight consecutive patients who underwent liver resection at our institution using the Saroa Surgical System between November 2024 and August 2025. During the initial implementation phase, patients with relatively superficial liver lesions that did not require anatomical resection were selected by the institutional surgical team as suitable candidates for the initial robotic approach. Patient characteristics and short‐term perioperative outcomes were retrospectively evaluated.

Ethical review was not required because this retrospective case series was determined to be outside the scope of the Ethical Guidelines for Medical and Biological Research Involving Human Subjects in Japan. Individual informed consent was not required because only sufficiently anonymized clinical information was used. All procedures were performed in accordance with domestic guidelines for the introduction of robotic liver surgery and the relevant proctoring standards established by Japanese academic societies.

### 2.2 Preoperative Assessment and Simulation

All patients underwent standard preoperative assessments, including blood tests, imaging studies, and fitness evaluations for surgery. Preoperative simulation was performed by the surgical team before each procedure, including reviews of three‐dimensional reconstruction images, operative strategy, patient positioning, port placement, and device selection.

### 2.3 Surgical Platform

The Saroa Surgical System was used in all cases. The surgeon console adopted an open‐platform design, allowing the use of visualization systems from other manufacturers as appropriate.

For visualization, either the 1788 system (Stryker, Kalamazoo, MI, USA) or VISERA ELITE III (Olympus, Tokyo, Japan) was used according to intraoperative requirements. Tumor identification and liver parenchymal transection were performed under indocyanine green fluorescence guidance when appropriate.

Robotic instruments designed for the Saroa Surgical System were used, including bipolar forceps, monopolar curved scissors, and a large‐needle driver (all Riverfield Inc., Tokyo, Japan). The bedside assistant used a Cavitron Ultrasonic Surgical Aspirator (CUSA Clarity, Integra LifeSciences, Princeton, NJ, USA) for liver parenchymal transection, an ultrasonic surgical device for division of small vessels (Sonicision, Medtronic, Minneapolis, MN, USA), a bipolar sealing device for hemostasis, and a laparoscopic suction‐irrigation system.

### 2.4 Force Feedback

Force feedback is a distinctive feature of the Saroa Surgical System. The system estimates the gripping force applied by the forceps from control information and relays this force to the surgeon at the remote‐control interface as pneumatic resistance perceived by the fingers. However, this feedback is limited to grasping force and does not reflect resistance generated by compression, pushing forces, or instrument‐to‐instrument interference. At the surgeon console, force‐feedback information is also displayed visually as quantitative values in newtons and as a bar graph, alongside the main endoscopic image.

### 2.5 Patient Position and Port Placement

**FIGURE 1:**
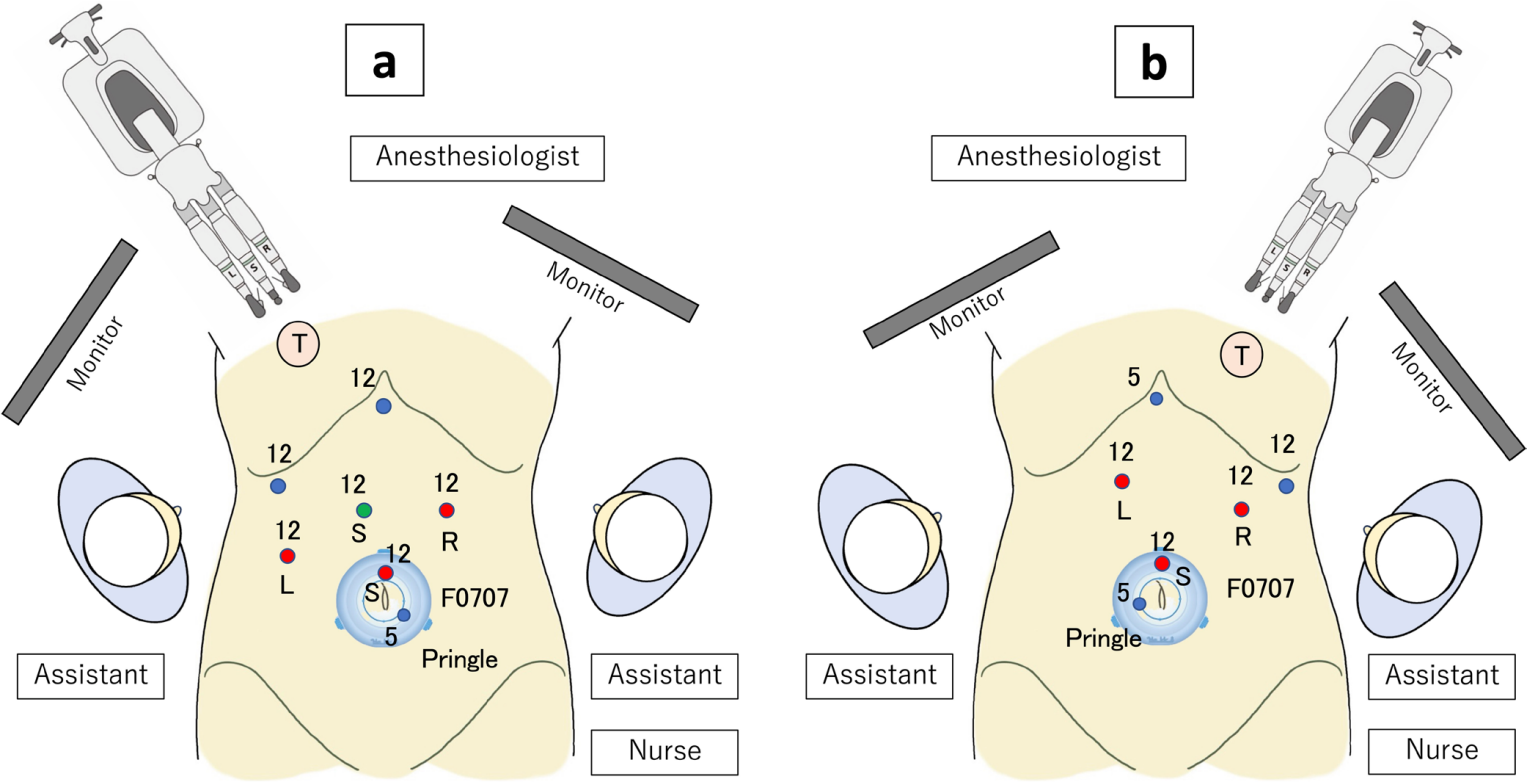
Operative setup and representative port placement. Representative operating room setup and port placement for (a) right‐ and (b) left‐sided liver lesions. Patients were placed in either the supine or left lateral decubitus position. The anesthesiologist was positioned at the patient's head, and two monitors provided visual guidance to the bedside assistants and scrub nurses. A 3.5‐cm transumbilical incision was made for the placement of an access device to accommodate a 12‐mm scope port and a 5‐mm port for the Pringle maneuver. For cranially located tumors, an additional scope port was inserted as needed (a). Two 12‐mm robotic instrument ports were placed bilaterally, and two assistant ports were positioned in the epigastric and lateral abdominal regions. L, left robotic instrument port; R, right robotic instrument port; S, scope port; T, target lesion.

The operative setup and representative port placement are shown in Figure 1. The patients were placed either in the supine or left lateral decubitus position, with both legs supported by a levitator system. Depending on the tumor location and operative requirements, the table was tilted up to 10° in the reverse Trendelenburg position and up to 10° laterally.

**FIGURE 2:**
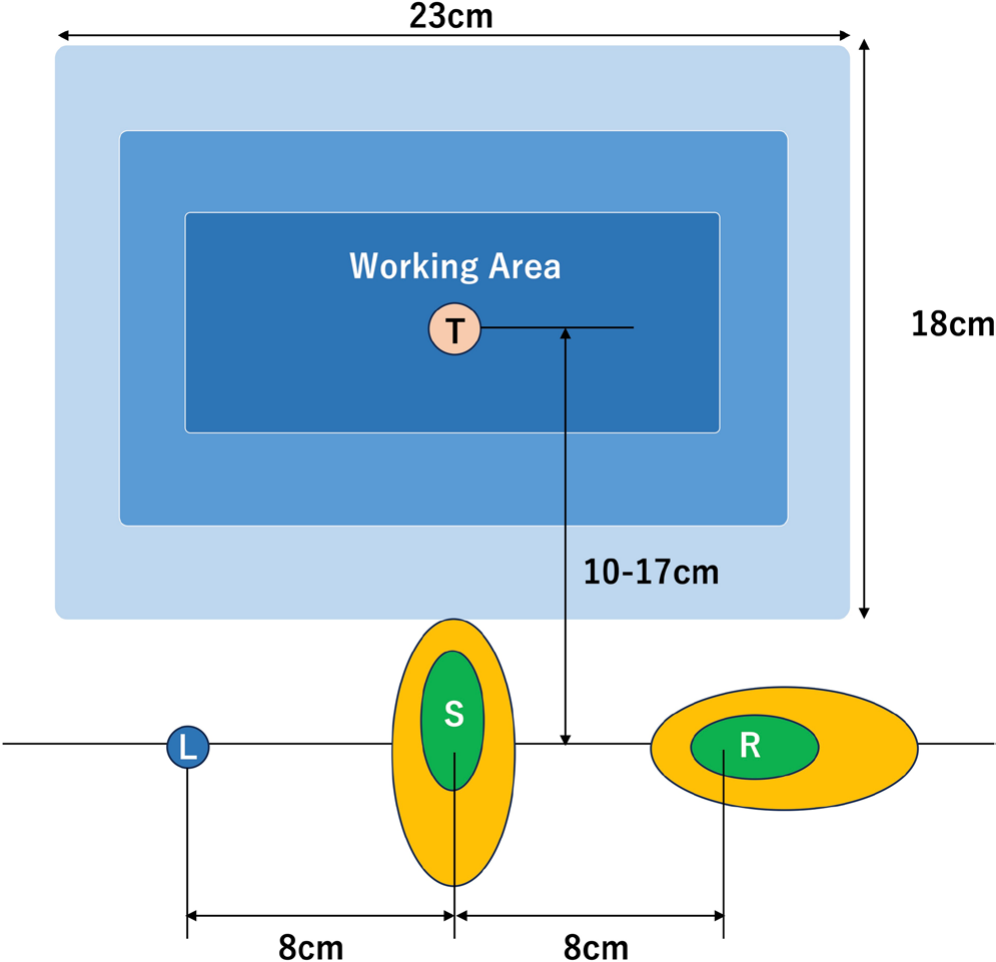
Port placement guide used for preoperative marking of robotic instrument ports. The schematic figure illustrates how the positions of the scope and robotic instrument ports were determined before skin incision. The distance between the line connecting the bilateral robotic ports and the target point was set at 10–17 cm, and the bilateral robotic ports were placed 8 cm from the scope port. L, left robotic instrument port; R, right robotic instrument port; S, scope port; T, target point.

To optimize access for the three robotic arms, the patient cart was positioned on the right side for right‐sided liver lesions (Figure 1a) and on the left side for left‐sided lesions (Figure 1b). The bedside assistant was positioned on both sides of the patient, the scrub nurse was positioned on the left side, and the anesthesiologist was positioned at the head of the patient. The two monitors provided visual information to the assistants and nurses. After induction of general anesthesia, the port sites were marked on the abdominal wall using a port placement guide sheet (Figure 2). Port placement was individualized according to tumor location, planned resection, and the presence of intra‐abdominal adhesions. A 3.5‐cm transumbilical incision was made, through which an access device (F0707, Hakko Co., Nagano, Japan) was inserted to accommodate a 12‐mm scope port and a 5‐mm port for the Pringle maneuver. An additional cranial scope port was used when required. Two 12‐mm robotic instrument ports and two assistant ports were inserted as shown in Figures 1 and 2.

### 2.6 Surgical Procedure

**FIGURE 3:**
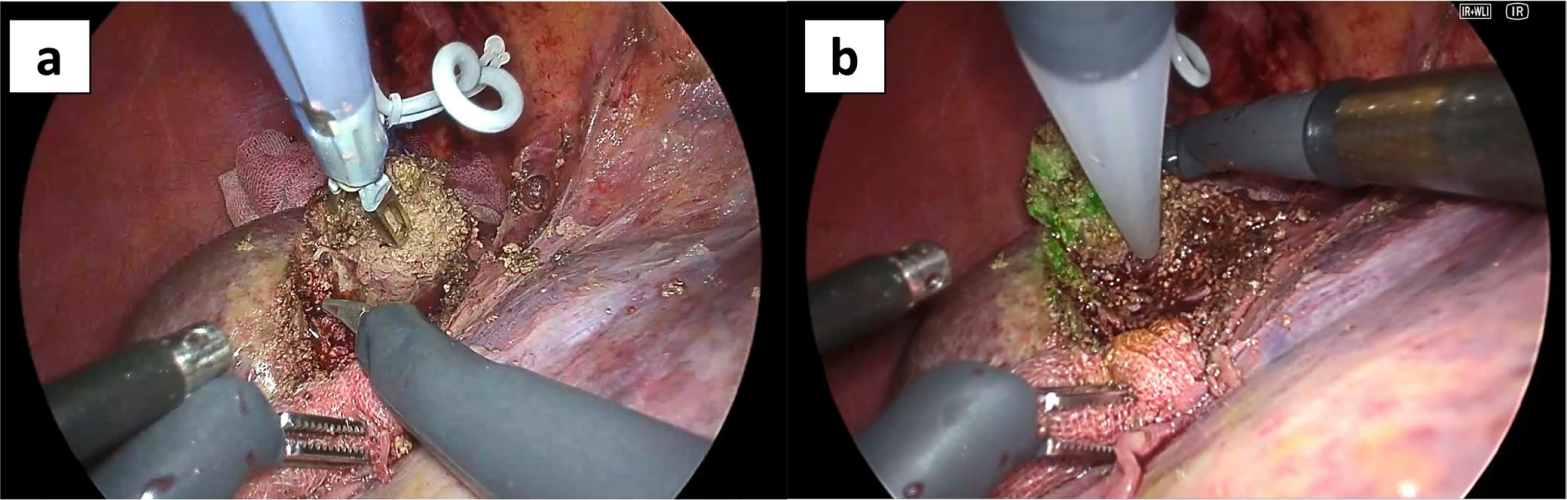
Representative intraoperative views of the two‐surgeon technique. During clamp‐crush parenchymal transection performed by the console surgeon, the bedside assistant provided operative exposure (a). Conversely, when the bedside assistant operated CUSA, the console surgeon provided operative exposure (b). CUSA, Cavitron Ultrasonic Surgical Aspirator.

Liver mobilization was performed laparoscopically, and intermittent inflow occlusion using an extracorporeal Pringle maneuver was routinely applied during parenchymal transection. Parenchymal transection was carried out using a two‐surgeon technique (Figure 3) [9]. The console surgeon applied countertraction with left‐hand forceps, while the clamp‐crush and SLiC‐Scissors techniques [10] were used as appropriate for parenchymal dissection. During deep parenchymal transection, the bedside assistant used CUSA or Sonicision when necessary.

During tissue handling, force feedback was provided at the surgeon's discretion. As the maximum grasping force of the Saroa forceps was relatively low (4 N), it was disabled during parenchymal transection when a greater grasping force was required. All procedures were performed by a single surgeon certified as an expert hepatobiliary‐pancreatic surgeon, a board‐certified endoscopic surgical specialist in Japan, and a da Vinci console surgeon.

### 2.7 Data Collection and Statistical Analysis

The clinical variables included patient characteristics, tumor‐related factors, operative procedure, and short‐term perioperative outcomes, including operative time, console time, hepatic inflow occlusion time, blood loss, conversion, postoperative complications, and postoperative hospital stay. Due to the limited sample size, no inferential statistical analysis was performed, and the data were presented descriptively.

## 3 Results

### 3.1 Patient Characteristics

Eight patients were included in the cohort, comprising six men and two women, with a median age of 75 years (range, 67–87 years). Patient characteristics are summarized in Table 1. The underlying diseases included hepatocellular carcinoma in five patients, metastatic liver tumors in two, and intrahepatic cholangiocarcinoma in one. The number of tumors ranged from one to two, and the tumor locations included segments 2, 3, 5, 6, 7, and 8. The median tumor size was 15 mm (range, 7–32 mm) and all patients underwent partial hepatectomy. In Patients 6 and 7, planned laparoscopic partial hepatectomy was performed for concomitant lesions in segments 5 and 2/3, respectively.

**TABLE 1:** Patient characteristics.

Case	Age	Sex	Disease type	BMI (kg/m^2^)	ASA‐PS	ICG‐R15	History of abdominal surgery	Tumor number	Tumor location	Tumor size (mm)	Operation	RLR combined with LLR
1	73	M	HCC	24.8	2	12.9	0	1	S8	32	PR	0
2	77	M	HCC	28.3	2	10.7	1	1	S6	15	PR	0
3	72	M	HCC	23.6	2	7.5	1	1	S8	15	PR	0
4	87	F	HCC	14.7	3	9.2	1	2	S2	20	PR	0
5	71	M	Liver metastasis (colon cancer)	20.0	2	8	1	1	S3	10	PR	0
6	67	F	Liver metastasis (P‐NEC)	28.5	2	8	1	2	S8, S5	13 (S8), 17 (S5)	PR (RLR), PR (LLR)	1
7	78	M	HCC	25.2	2	15.9	0	2	S6/7, S2/3	15 (S6/7), 15 (S2/3)	PR (RLR), PR (LLR)	1
8	81	M	ICC	24.1	2	1.4	0	1	S3	20	PR	0

Abbreviations: ASA‐PS, American Society of Anesthesiologists physical status; BMI, body mass index; F, female; HCC, hepatocellular carcinoma; ICC, intrahepatic cholangiocarcinoma; ICG‐R15, indocyanine green retention rate at 15 min; LLR, laparoscopic liver resection; M, male; P‐NEC, pancreatic neuroendocrine carcinoma; PR, partial liver resection; RLR, robotic liver resection.

### 3.2 Surgical Outcomes

The operative parameters and short‐term perioperative outcomes are summarized in Table 2. The median operative, console, and hepatic inflow occlusion times were 267 min (range, 212–354 min), 136 min (range, 74–179 min), and 41 min (range, 0–82 min), respectively. The median blood loss was 17.5 mL (range, 10–170 mL). None of the patients required conversion to open surgery, whereas three patients underwent unplanned conversion to laparoscopy. In two patients, conversion was performed because a prolonged Pringle maneuver time was anticipated, whereas in one patient, severe adhesions precluded a safe operative view with the robotic approach. One patient who underwent a third hepatectomy developed postoperative bile leakage (Clavien–Dindo grade IIIa). The median postoperative hospital stay was 10 days (range, 8–30 days).

**TABLE 2:** Operative parameters and short‐term perioperative outcomes.

Case	Operative time (min)	Console time (min)	HIO time (min)	Blood loss (mL)	Resected liver weight (g)	Laparoscopic conversion	Reason for conversion	Postoperative complications	Complication details	LOS (days)
1	304	169	64	50	34.1	1	Prolonged Pringle time	0		10
2	270	179	47	10	14.4	0		0		8
3	240	148	28	10	5.0	0		0		10
4	342	74	0	170	7.2	1	Difficulty in achieving a safe view	1	Bile leakage (Clavien–Dindo IIIa)	30
5	212	135	35	10	7.6	0		0		8
6	263	75	18	70	10.8 (RS), 3.0 (LS)	0		0		8
7	354	136	82	25	10.3 (RS), 7.8 (LS)	0		0		10
8	253	81	56	10	13.6	1	Prolonged Pringle time	0		14

Abbreviations: HIO, hepatic inflow occlusion; LOS, postoperative length of hospital stay; LS, laparoscopic specimen; RS, robotic specimen.

## 4 Discussion

Robotic liver resection has been increasingly adopted in recent years, with clinical experience reported using several surgical platforms, including the da Vinci Surgical System [11], the da Vinci SP system [12], the hinotori Surgical Robot System [13], and the Hugo RAS system [14]. To the best of our knowledge, this is the first case series describing robotic liver resection using the Saroa Surgical System. Our initial experience suggests that Saroa may represent a practical platform for liver resection. At the same time, this series revealed several unique advantages, including compatibility with the laparoscopic workflow, as well as current technical challenges that should be addressed through further system refinement.

Concerning perioperative safety, one patient developed postoperative bile leakage (Clavien–Dindo grade IIIa). The patient had a biliary stricture requiring regular biliary tube exchange, which may have influenced the postoperative biliary course. Notably, none of the patients required conversion to open surgery. Although three patients required unplanned conversion to laparoscopy, the Saroa Surgical System's port configuration, which is compatible with laparoscopic surgery, enabled conversion without major disruption to the operative workflow. This feature offers a significant practical advantage, particularly during the initial implementation phase.

A practical strength of Saroa is its compact and low‐cost design [15]. Unlike larger robotic platforms [11, 13], it does not require major modifications to the operating room environment and can be integrated into an existing laparoscopic setup. This may lower the institutional barriers to adoption and make the system particularly suitable for centers seeking stepwise implementation of robotic liver surgery, especially those with lower case volumes.

From a technical perspective, Saroa combined articulated instrument movement with force feedback, which may be advantageous for delicate tissue handling in liver surgery [16]. In our series, no obvious collateral injury attributable to excessive grasping force was observed. Although this finding does not establish causality, the combination of force feedback and reduced maximum grasping force may have contributed to atraumatic tissue handling [17]. However, the maximum grasping force of the Saroa forceps is relatively low at 4 N, approximately half that of the da Vinci system, and clamp‐crush parenchymal transection is occasionally difficult, particularly in severely fibrotic or cirrhotic liver parenchyma. To address this issue, shorter Maryland forceps were adopted, increasing grasping force to approximately 5–6 N, and force feedback was selectively disabled when a greater grasping force was required. These findings underscore both the promise and the current limitations of force feedback in robotic liver resection. The force‐feedback capability of Saroa may facilitate gentle traction and controlled tissue manipulation, partially offsetting the predominantly visual nature of robotic surgery. However, the limited maximum grasping force of the current forceps may constrain its utility during parenchymal transection, particularly in fibrotic or cirrhotic liver. Further device refinements are therefore needed to translate this potential into consistent clinical benefit.

Another distinctive feature of the Saroa system is its three‐arm, docking‐free design, which creates more working space around the patient and facilitates the bedside assistant's active participation. Existing platforms that use multiple robotic arms in combination with robot‐specific ports [11, 12, 13, 14] are generally structured around a console‐surgeon‐centered operative concept. By contrast, the present configuration may be more amenable to team‐based surgery, including a two‐surgeon operative approach [9]. In our series, planned laparoscopic partial hepatectomy for additional lesions was intentionally performed by trainees in two patients, suggesting a possible educational role of this platform in stepwise liver surgery training [18]. However, this potential advantage remains speculative and should be evaluated in future studies.

Our initial experience highlighted several limitations of this system. First, the working range was narrower than that of established robotic platforms, and the instrument length was relatively short (approximately 35 cm). These factors occasionally limited access to the posterosuperior segments, liver mobilization, and taping of the hepatoduodenal ligament. In addition, slight instability related to the pivot‐free design was occasionally observed during manipulation. Furthermore, the limited availability of dedicated robotic instruments, such as suction devices, clips, and energy devices, remains a practical drawback. In this series, assistant‐supported transection using laparoscopic devices, including CUSA and Sonicision, played an important complementary role during parenchymal dissection. Further technological refinement and expansion of the dedicated instrument lineup are necessary to optimize Saroa's role in liver surgery.

This study had several limitations. This was a retrospective case series from a single institution and included a small number of selected patients with relatively superficial lesions who underwent partial hepatectomy during the initial implementation phase of the Saroa system. All procedures were performed by a single, highly experienced hepatobiliary surgeon, which may have influenced the perioperative outcomes. In addition, no direct comparisons with conventional laparoscopic surgery or other robotic platforms were performed. Therefore, the generalizability of our findings is limited. Nevertheless, our experience suggests that the Saroa Surgical System may be a practical option for robotic liver resection, particularly in settings where compatibility with the laparoscopic workflow, ease of implementation, and team‐oriented training are valued. Further clinical experience and technological refinements are required to clarify its role in hepatobiliary surgery.

In conclusion, the Saroa Surgical System appears to be a practical platform for robotic liver resection in selected patients. Its compatibility with the laparoscopic workflow, compact design, and force‐feedback capability may offer practical advantages during early implementation, with potential relevance to stepwise surgical training. However, technical limitations, including a restricted working range, limited instrument availability, and relatively low grasping force, remain to be addressed. Further clinical experience and technological refinements are required to clarify its role in liver surgery.

## Disclosure

All authors made substantial contributions to the study, revised the manuscript critically for important intellectual content, approved the final version of the manuscript, and agreed to be accountable for all aspects of the work.

## Conflicts of Interest

The authors declare no conflicts of interest.

## Data Availability

The datasets generated and/or analyzed during the current study are not publicly available due to privacy and ethical restrictions.
